# Artificial intelligence tools for the identification of antibiotic resistance genes

**DOI:** 10.3389/fmicb.2024.1437602

**Published:** 2024-07-12

**Authors:** Isaac Olatunji, Danae Kala Rodriguez Bardaji, Renata Rezende Miranda, Michael A. Savka, André O. Hudson

**Affiliations:** ^1^Thomas H. Gosnell School of Life Sciences, College of Science, Rochester Institute of Technology, Rochester, NY, United States; ^2^School of Chemistry and Materials Science, College of Science, Rochester Institute of Technology, Rochester, NY, United States

**Keywords:** artificial intelligence, antibiotic resistance, antibiotic resistance genes, deep learning, Hidden Markov Model, support vector machines, random forest

## Abstract

The fight against bacterial antibiotic resistance must be given critical attention to avert the current and emerging crisis of treating bacterial infections due to the inefficacy of clinically relevant antibiotics. Intrinsic genetic mutations and transferrable antibiotic resistance genes (ARGs) are at the core of the development of antibiotic resistance. However, traditional alignment methods for detecting ARGs have limitations. Artificial intelligence (AI) methods and approaches can potentially augment the detection of ARGs and identify antibiotic targets and antagonistic bactericidal and bacteriostatic molecules that are or can be developed as antibiotics. This review delves into the literature regarding the various AI methods and approaches for identifying and annotating ARGs, highlighting their potential and limitations. Specifically, we discuss methods for (1) direct identification and classification of ARGs from genome DNA sequences, (2) direct identification and classification from plasmid sequences, and (3) identification of putative ARGs from feature selection.

## Background

When antibiotics were first discovered in the early twentieth century, it marked a monumental shift in the battle against bacterial infections. The journey of antibiotic research and development was paved with many years of incremental progress, from the initial observations of bacteria structure by Antonie van Leeuwenhoek to the recognition of mold’s curing abilities by John Parkington in the seventeenth century to the disproof of the abiogenesis theory, and the characterization of infectious diseases (Mohr, 2016). The discovery of penicillin by Fleming (1929) and its subsequent mass production at the United States Department of Agriculture (USDA) Northern Regional Research Laboratory in Peoria, Illinois, was a turning point that saved tens of thousands of men in the Second World War from wound infections. This breakthrough was swiftly followed by the introduction of several antibiotics in the next decades (Kourkouta, 2018). In the following years, many antibiotics were developed, each with its unique mode of action (Baran et al., 2023).

Unfortunately, due to the lack of stewardship regarding the use of antibiotics and the natural process of evolution, bacteria have circumvented the efficacy of clinically relevant antibiotics, leading to antibiotic resistance. The gravity of this situation cannot be overstated. It should be highlighted that antibiotic resistance was noticed almost immediately after penicillin was discovered. Fleming had observed as early as 1929 “*that the growth of E. coli and a number of other bacteria belonging to the coli-typhoid group was not inhibited by penicillin*” (Mohr, 2016). He attributed it to inaccurate dosage. Later experiments using *E. coli* by Abraham and Chain would come to reveal that, in fact, an enzyme produced by the bacteria was quashing the bacterial growth-inhibiting property of penicillin (Abraham and Chain, 1940; Mohr, 2016). Streptomycin was ushered in 1944 for the treatment of tuberculosis. In response, resistant variants of *Mycobacterium tuberculosis* were soon detected. Even scarier was the revelation in Japan that resistance abilities could be transferred vertically and horizontally across bacteria populations through plasmid transfer, and the subsequent identification of multidrug-resistant bacteria was identified in the 1960s. This recurrent sequence of new antibiotics discovery and rapid bacteria resistance development has been the normal sequence of events to date (Davies and Davies, 2010; Podolsky, 2018). According to the Centers for Disease Control and Prevention, the threat of antibiotic resistance is a global public health emergency. This crisis currently results in 2.8 million infections in the United States, leading to approximately 35,000 deaths annually because of antibiotic resistance (Dadgostar, 2019). The annual estimated cost of treating six common multidrug-resistant bacterial illnesses was around $4.6 billion (Center for Disease Control and Prevention, 2021; Nelson et al., 2021). Other recognized burdens of antibiotic resistance include severe illnesses, increased length of hospital stay, and complete treatment failure. There is an antibiotic resistance crisis, and urgent steps are needed to avert a return to the pre-antibiotic era (Martens and Demain, 2017).

Based on currently available antibiotics, several strategies have been proposed for combating this crisis: the development of new antibiotics, phage therapy, combination therapy, antibody therapy, immune modulation, and the One Health approach, among others (Muteeb et al., 2023). It is especially critical to double down on efforts toward innovating and developing new antibiotics as work in this area has recently slowed. There are reported cases of bacterial resistance to last-resort drugs, like *Klebsiella* and carbapenem, which expose the population to the risk of untreatable infections. There is projected to be a two-fold increase in resistance to last-resort antibiotics compared to the 2005 level (WHO, 2023). The increased costs of research and development, coupled with the lack of incentives, have made the thrust for the research and development of infectious diseases an unattractive pursuit to pharmaceutical companies (Piddock, 2012; Ventola, 2019; Muteeb et al., 2023).

Direct inactivation of drugs, limit in drug uptake, modification of drug target, and increase in active drug efflux pumps are well-known modes of action by which bacteria resist antibiotics. However, the basis for these modes of action usually can be traced back to genetic mutations and antibiotic resistance genes (ARGs), which are often localized on plasmids and thus transferable between various bacterial genera, species, or strains (Muteeb et al., 2023). It is necessary to identify and understand these mutations and ARGs that can serve as viable targets at various levels for new antibiotic compounds and help to understand antibiotic resistance transmission better (Hughes and Karlén, 2014). Currently, existing computational workflows for identifying ARGs from next-generation sequencing (NGS) data are mostly based on assembly or read-based methods, which rely on sequence alignment for mapping reads to the genome. These are limited in their ability to identify new ARGs and are prone to false positives due to reading similarity in the read-based methods (Hunt et al., 2017; Lakin et al., 2017; Yin et al., 2018; Alcock et al., 2020). Emerging AI-based methods promise to overcome some of these hurdles. Machine learning (ML) and deep learning (DL) are subfields of AI. DL models can extract features from known ARG sequences and use these to identify novel ARGs (Lakin et al., 2017; Roy et al., 2023). ML algorithms continuously learn new information from datasets without being explicitly programmed, and deep learning employs layers of neural networks that mimic human neurons to learn novel information from a dataset. Depending on the task, both algorithms can be grouped as supervised or unsupervised learning, and DL algorithms can be further classified into reinforcement learning (Farina et al., 2022; Ali et al., 2023; Vodanović et al., 2023). In the biomedical field, AI is a great resource for making sense of the enormous data generated from high-throughput molecular technologies (NHGRI, 2022). In this review, we summarize the application of AI for the identification and annotation of ARGs.

## Identification and annotation of antibiotic resistance genes (ARGs)

Traditional methods for identifying ARGs from NGS data consist of mapping reads directly to a reference genome or assembling the reads into contigs before being compared to the reference database. These methods cannot identify novel ARG sequences and are often limited by false negative and false positive results (Chowdhury et al., 2020; Wu et al., 2023). They are also unable to distinguish between chromosomal or plasmid sequences. AI models now exist to identify ARGs directly from short NGS raw reads or fully assembled genes (Hunt et al., 2017; Lakin et al., 2017; Yin et al., 2018; Alcock et al., 2020). Studies have also applied AI to identify tentative ARGs and validate them. Encouragingly, some of these models have recorded metrics comparable to the strict alignment methods (Lakin et al., 2017; Roy et al., 2023). Common ARG identification and annotation algorithms include support vector machine (SVM), neural networks, and Hidden Markov Models (HMM). The feature selection component in eXtreme Gradient Boosting (XGBoost) and random forest (RF) are useful in identifying potential ARGs.

**FIGURE 1:**
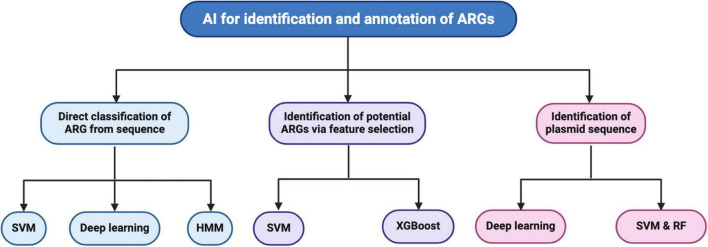
Summary of machine learning and deep learning algorithms for identifying antibiotic resistance genes (ARGs) and plasmids described in this review. Studies reviewed were under the direct classification of ARGs from genome sequences utilizing support vector machine (SVM), deep learning, and Hidden Markov Model (HMM) algorithms. The reviewed studies utilized SVM and eXtreme Gradient Boosting (XGBoost) to identify potential ARGs via feature selection. Deep learning, SVM, and RF were utilized in studies to identify plasmid sequences.

Here, we discuss studies on the direct classification of ARGs from sequence data, identifying possible ARGs from sequence data via feature selection, and identifying plasmid sequences. Figure 1 illustrates these three approaches: machine learning, deep learning algorithms for identifying antibiotic resistance genes (ARGs), and plasmids.

## Direct classification for identification of ARGs from sequence data

The studies are presented based on the types of model algorithms. The tools described in this review along with the hyperlinks to these tools are annotated in Table 1.

**TABLE 1:** ARG and plasmid identification tools discussed in this review and their weblinks.

Tool	Algorithm	ARG or plasmid identification	References	Weblink
BlaPred	SVM	ARG	Srivastava et al., 2018	http://proteininformatics.org/mkumar/blapred
BacEffluxPred	SVM	ARG	Pandey et al., 2020	http://proteininformatics.org/mkumar/baceffluxpred/
MP4	SVM	ARG	Gupta et al., 2022	http://metagenomics.iiserb.ac.in/mp4
mlplasmids	SVM	Plasmid	Arredondo-Alonso et al., 2018	https://gitlab.com/sirarredondo/mlplasmids
DeepARG	Deep learning	ARG	Arango-Argoty et al., 2018	http://bench.cs.vt.edu/deeparg
PLM-ARG	Deep learning	ARG	Wu et al., 2023	https://github.com/Junwu302/PLM-ARG
HMD-ARG	Deep learning	ARG	Li et al., 2021	http://www.cbrc.kaust.edu.sa/HMDARG/
ARG-SHINE	Deep learning	ARG	Wang et al., 2021	https://github.com/ziyewang/ARG_SHINE
PlasFlow	Deep learning	Plasmid	Krawczyk et al., 2018	https://github.com/smaegol/PlasFlow
Deeplasmid	Deep learning	Plasmid	Andreopoulos et al., 2022	https://github.com/wandreopoulos/deeplasmid
PPR-META	Deep learning	Plasmid	Fang et al., 2019	https://github.com/zhenchengfang/PPR-Meta
PlansTrans	Deep learning	Plasmid	Fang and Zhou, 2020	https://github.com/zhenchengfang/PlasTrans
Meta-MARC	HMM	ARG	Lakin et al., 2019	https://github.com/lakinsm/meta-marc-publication/blob/master/analytic_data/mmarc_test_set.fasta
SurHMM	HMM	ARG	Xie and Fair, 2021	https://gitlab.com/gary_xie/surhmms
SourceFinder	RF	Plasmid	Aytan-Aktug et al., 2022	https://cge.food.dtu.dk/services/SourceFinder/

ARG, antibiotic resistance genes; DL, deep learning; HMM, Hidden Markov Model; RF, random forest; SVM, support vector machine.

### Support vector machines (SVM)

β-lactams are the largest group of antibiotics that are employed in a clinical setting, and it is no surprise that β-lactamases are the most common form of resistance posed by bacteria against antibiotics. With various chemical modifications of β-lactams introduced over the years, bacteria have also evolved in the types of β-lactamases produced. It is, therefore, important to accurately characterize β-lactamase to administer the right therapy. An algorithm SVM-based model was created to fast track this tedious and time-consuming laboratory process (Srivastava et al., 2018). SVM is a machine learning-based classification algorithm well known for its robustness to outliers and ability to deal with high-dimensional datasets, frequently encountered in bioinformatics (Van Messem, 2020). Multi-level SVM models developed in this study take in protein sequences represented in the form of amino acid composition (AAC) or pseudo amino acid composition (PseAAC) as described in Chou (2001, 2005) and classify β-lactamase A, B, C, or D and if B, further into B1, B2, B3. Validated using a leave one out cross validation (LOOCV), PseAAC input models performed better, with accuracy scores ranging from 82 to 97%. In a separate study, the Srivastava group applied SVM models to tackle another antibiotic resistance mechanism- efflux pump proteins. BacEffluxPred, a two-level group of SVM models, was developed to identify and classify bacterial efflux pump proteins into various families (Pandey et al., 2020). The level I model for distinguishing antibiotic resistance efflux (ARE) protein from non-AREs achieved an accuracy score of 85 and 94% on the training and independent datasets, respectively. Level II model achieved an accuracy score of 93, 93, 93, and 100%, respectively, in a LOOCV when each of the four ARE classes- ATP binding cassette (ABC) transporter, major facilitator superfamily (MFS), small multidrug resistance (SMR), multidrug and toxic compound extrusion (MATE) families were grouped against a combined group of other three classes.

An SVM model performed best to differentiate between pathogenic and non-pathogenic bacterial proteins from sequences represented as dipeptide frequency and pepstatin-containing vectors, recording 79 and 72% accuracy on two separately curated datasets, respectively (Gupta et al., 2022). The pathogenic proteins were grouped into three, with one of the groups containing ARGs and toxins.

### Deep learning (DL)

Perhaps the most recognized deep learning-based ARG identification system currently is DeepARG (Arango-Argoty et al., 2018), a collection of artificial neural network (ANN) models- DeepARG-LS and DeepARG-SS for identifying ARGs directly from assembled sequences and short reads, respectively. Trained on DeepARG-DB, a manually curated database put together from CARD (Jia et al., 2017), ARDB (Liu and Pop, 2009), and UniProt (Apweiler et al., 2004), the models have recorded precision and recall scores above 0.97 and 0.90, respectively. Protein sequences for training the models were represented as N * 4333 vector matrices containing bit scores (similarity distance between UniProt training sequences and known sequences in CARD and ARDB) and passed through the ANNs to predict 30 classes of antibiotic resistance, including “unknown” class. Similarly, a Large Language Model (LLM), ESM-1b, originally trained on about 250 million protein sequences (Rives et al., 2021), was combined with XGBoost to identify ARGs and classify their resistance group. PLM-ARG, as it is named by the authors, embedded protein sequences with ESM-1b and trained the XGBoost models on the embedding to identify ARGs and classify ARGs resistance (Wu et al., 2023). On an independent test dataset, PLM-ARG recorded metrics ranging from 9.6 to 36% and 40.8 to 107.3%, respectively, in AUC and f1-scores above RGI, ResFam and DeepARG, three other state-of-the-art (SOTA) ARG prediction methods.

Various categorizations of ARGs exist to enable a better understanding of the spread of antibiotic resistance and ecology. Beyond the identification of ARGs, (Li et al., 2021) built a hierarchical multi-task deep learning framework for ARG annotation (HMD-ARG), a Convolutional Neural Network (CNN) based system that classifies ARGs at different levels. The model takes a raw protein sequence that is one hot encoded and, in downward order, predicts if the sequence is an ARG, the antibiotic class it is resistant, its resistance mechanism (mode of action), whether the ARG is intrinsic or an acquired, and what subclass of β-lactamase it belongs to if it is a β-lactamase. Manually curated sequences from seven databases- CARD (Jia et al., 2017), AMRFinder (Feldgarden et al., 2019), ResFinder (Zankari et al., 2012), ARG-ANNOT (Gupta et al., 2014), DeepARG (Arango-Argoty et al., 2018), MEGARes (Lakin et al., 2017), and Resfams (Gibson et al., 2015) were labeled according to 15 antibiotic resistance classes in addition to the 6 mechanisms of antibiotic resistance (enzyme inactivation, modified target, resistance-conferring plasmid, modified cell wall/membrane, efflux pumps overexpression and resistance mutations), and gene transferability. In all tasks, an accuracy score of greater than 0.9 was recorded, and experimental validation of 8 randomly selected genes from *Pseudomonas aeruginosa* agreed with HMD-ARG model predictions. For ARG classification, another method ensembled ARG-CNN that is based on CNN classification of sequence embedding, ARG-InterPro is based on logistic regression classification of protein domains, families, and functional sites data, and ARG-KNN is based on K-nearest neighbor (KNN) classification of BLAST alignment homology results to classify ARGs (Wang et al., 2021). The resulting overall model named ARG-SHINE outperformed other known ARG classification methods, including BLAST best hit (Altschul et al., 1990), DIAMOND best hit (Buchfink et al., 2014), DeepARG (Arango-Argoty et al., 2018), HMMER (Eddy, 2011), and TRAC (Hamid, 2019).

Manually curated ARG databases like CARD (Jia et al., 2017) utilized text-mining algorithms in ranking publications for manual review. Taking advantage of Natural Language Processing to incorporate deep learning into this process can lead to further improvements. A Biomedical Relation Extraction (BioRE) system trained on PubMed, CARD (Jia et al., 2017),and UniProtKB (Apweiler et al., 2004) datasets at the sentence level were built to predict gene-antibiotic relations that can be useful to further enhance the process of ARG curation from publications (Brincat and Hofmann, 2022). BioBERT, a transformer-based model trained on biomedical data, and Piecewise Convolution Neural Network (PCNN) were trained separately on the datasets. BioBERT performed best on the holdout test dataset and was used to identify gene-antibiotic relations for metronidazole in *H. pylori*.

### Hidden Markov Models (HMM)

Annotated genes from the MEGARes database (Lakin et al., 2017) were clustered according to sequence similarity, and an HMM was trained on each gene cluster to produce multiple HMM models that classify an input sequence as ARG and predict its origin (Lakin et al., 2019). The model recorded high mean sensitivity and specificity scores between 97 and 99%. Xie and Fair (2021) combined the identification of unique family substring true and junction markers characteristics of Short Better Representative Extract Dataset (ShortBRED) with HMMs that are based on these markers for accurate identification of bacterial toxins, virulence factors, and antimicrobial resistance sequences from NGS reads.

## Features selection methods for identification of potential ARGs

Features selection is a dimensionality reduction technique that allows for selecting the most relevant variables that produce the best prediction results from many variables. As it applies to ARGs, selecting the most important ARGs with machine learning for prediction tasks relevant to antibiotic resistance could lead to identifying novel ARGs. Features selection methods can generally be filter, wrapper, or embedded. Alongside embedded methods like decision tree-based XGBoost and random forest, we found from the literature that the SVM algorithm, which falls within the wrapper feature selection group, is one of the most commonly employed algorithms for identifying potential ARGs. SVM was combined with a recursive feature addition function for identifying genes and mutations associated with resistance to pyrazinamide, a common antibiotic for treating *Mycobacterium tuberculosis* infection (Zhang et al., 2021). Trained on the binary representation of mutations on 23 resistance-related genes for the bacteria strains included in the study (Zhang et al., 2021), the model identified three likely ARGs–embB, gyrA, and pncA, which contain 104 unique mutations associated with Pyrazinamide resistance, one (pncA) of which is already known. Prediction of Pyrazinamide resistance with the 104 mutations led to an accuracy score of 89%. To further verify the novelty of the two unknown genes as resistant to Pyrazinamide, mutations on only the two genes were used as features for predicting pyrazinamide resistance. An accuracy of 72% was achieved.

Support vector machine-random subspace ensembles (SVM-RSEs) consist of multiple SVM models, each built from randomly selected 80% of samples and 50% of features (Hyun et al., 2020). In the end, features were ranked by weight. Pan genome was constructed from genomes of *Staphylococcus aureus*, *Escherichia coli*, and *Pseudomonas aeruginosa* downloaded from PATRIC (Wattam et al., 2014), and binary representations of the genomes were imputed into the models to predict antibiotic susceptibility. This technique identified more known ARGs than Fisher’s Exact and Cochran-Mantel-Hanszel (CMH) statistical tests, and it recorded an accuracy score of 79 to 99% and AUC of 0.79 to 1.0.

A game theory-based feature selection technique was combined with an SVM classifier to select the best overall representative features that predict ARGs (Chowdhury et al., 2019). The technique is based on weights assigned to a newly selected feature, which depends on weights of already selected features, followed by overall weights readjustment, described as the interdependence of the selected features. From an initial size of 621 features comprising amino acid composition, physicochemical characteristics, and evolutionary and structural information, the selected features recorded an overall accuracy ranging from 91 to 99% in predicting ARGs.

Feature selection component of decision tree based algorithms have also been applied for identifying ARGs. ARGs selected via the XGBoost method from the binary pan genome representation of *A. baumannii*, *E. coli, K. pneumonia*, and *S. aureus* lead to a better antibiotic susceptibility prediction compared to models of already known AMR genes, all genes, or scary-selected genes (Yang and Wu, 2022). RF algorithm identified three virulence genes- racR, ceuE, pldA that are related to antibiotic susceptibility in *Campylobacter jejuni*, and *coli* species, in a study that was aimed at unraveling the poorly understood relationship between bacteria virulence and antibiotic resistance (Gharbi et al., 2022). Likewise, multiple ML models were trained to predict antibiotic resistance from all annotated genes in the NCBI database, and potential ARGs selected from the models were further validated by predicting their structure via homology modeling (Srivastava et al., 2018). It was observed that proteins coded by these unknown ARGs have higher binding affinity to antibiotics than known AMR proteins and randomly selected proteins. Nevertheless, the results and the need for expert guidance were rightly noted, as not all ARGs work by interacting directly with antibiotics (Muteeb et al., 2023).

## Identification of plasmid sequence

Bacterial plasmids carry genetic elements that can be transferable. These sequences typically differ from chromosomal elements and encode proteins such as ARGs, ensuring survival in a dynamic environment. Identification of plasmid sequences can indirectly lead to identifying ARGs and a deeper understanding of the potential spread of plasmids that carry ARGs. Differentiating plasmid sequences from chromosomal sequences has been challenging due to either assembly or incomplete databases. Examples of deep learning methods developed for the identification and differentiation of plasmid from chromosomal sequences include PlasFlow (Krawczyk et al., 2018), Deeplasmid (Andreopoulos et al., 2022), and PPR-Meta (Fang et al., 2019). PlansTrans was developed based on CNN to distinguish between transmissible and non-transmissible plasmids (Fang and Zhou, 2020). Arredondo-Alonso developed an SVM-based classifier to identify plasmids and predict ARG location in *E. faecum*, *K. pneumoniae*, and *E. coli* (Arredondo-Alonso et al., 2018). An RF classifier differentiated between plasmid, chromosomal, and bacteriophage sequences in assembled metagenomic datasets (Aytan-Aktug et al., 2022). The best-performing model achieved accuracy scores of 0.97, 0.94, and 0.93 per class for chromosome, plasmid, and bacteriophage sequences, respectively, and model performance was affected by the size of the k-mer (nucleotide sequence of a certain length) used for sequence representation.

## Limitations and conclusion

The application of AI undoubtedly has a place in the fight against antibiotic resistance, specifically for identifying and annotating ARGs, as shown in the reviewed publications. However, AI’s challenges and limitations to these tasks must be acknowledged to channel and maximize its utility effectively. While systems like DeepARG have demonstrated the ability to identify ARGs from short sequences, better results are obtainable from already assembled genes containing more sequence information. On the other hand, the computational resources and time required for running an assembler before predicting via DeepARG must also be considered (Arango-Argoty et al., 2018). Although the ARG proteins identified in the Sunuwar and Azad (2022) study are bound to antibiotics with high affinities, not all ARGs directly interact with antibiotics. In addition, ARGs identified by AI methods still require non-computational experimental validation (Arango-Argoty et al., 2018; Sunuwar and Azad, 2022). This further re-emphasizes that AI can be used as a tool and guided by domain experts to help address the problem of antibiotic resistance.

The supervised learning technique employed by many studies for identifying and classifying ARGs are limited by the spectrum of the labels assigned to data pre-model training. Therefore, it cannot recognize entities that fall outside the assigned labels (Arango-Argoty et al., 2018). Relatedly, the need and dearth of high quality, well-curated datasets in the biomedical space is a challenge that must be addressed soon to maximize the potential of AI (Zhang et al., 2021; Brincat and Hofmann, 2022). The studies reviewed here focus more on ARGs and do not specifically address intrinsic mutations associated with antibiotic resistance (Wu et al., 2023).

## Author contributions

IO: Writing – original draft, Writing – review & editing. DB: Writing – original draft, Writing – review & editing. RM: Writing – original draft, Writing – review & editing. MS: Writing – original draft, Writing – review & editing. AH: Funding acquisition, Writing – original draft, Writing – review & editing.
